# A microsatellite-based analysis for the detection of selection on BTA1 and BTA20 in northern Eurasian cattle (*Bos taurus*) populations

**DOI:** 10.1186/1297-9686-42-32

**Published:** 2010-08-06

**Authors:** Meng-Hua Li, Terhi Iso-Touru, Hannele Laurén, Juha Kantanen

**Affiliations:** 1 Biotechnology and Food Research, MTT Agrifood Research Finland, FI-31600 Jokioinen, Finland

## Abstract

**Background:**

Microsatellites surrounding functionally important candidate genes or quantitative trait loci have received attention as proxy measures of polymorphism level at the candidate loci themselves. In cattle, selection for economically important traits is a long-term strategy and it has been reported that microsatellites are linked to these important loci.

**Methods:**

We have investigated the variation of seven microsatellites on BTA1 (*Bos taurus *autosome 1) and 16 on BTA20, using bovine populations of typical production types and horn status in northern Eurasia. Genetic variability of these loci and linkage disequilibrium among these loci were compared with those of 28 microsatellites on other bovine chromosomes. Four different tests were applied to detect molecular signatures of selection.

**Results:**

No marked difference in locus variability was found between microsatellites on BTA1, BTA20 and the other chromosomes in terms of different diversity indices. Average *D*' values of pairwise syntenic markers (0.32 and 0.28 across BTA 1 and BTA20 respectively) were significantly (*P *< 0.05) higher than for non-syntenic markers (0.15). The Ewens-Watterson test, the Beaumont and Nichol's modified frequentist test and the Bayesian *F*_ST_-test indicated elevated or decreased genetic differentiation, at *SOD1 *and *AGLA17 *markers respectively, deviating significantly (*P *< 0.05) from neutral expectations. Furthermore, lnRV, lnRH and lnRθ' statistics were used for the pairwise population comparison tests and were significantly less variable in one population relative to the other, providing additional evidence of selection signatures for two of the 51 loci. Moreover, the three Finnish native populations showed evidence of subpopulation divergence at *SOD1 *and *AGLA17*. Our data also indicate significant intergenic linkage disequilibrium around the candidate loci and suggest that hitchhiking selection has played a role in shaping the pattern of observed linkage disequilibrium.

**Conclusion:**

Hitchhiking due to tight linkage with alleles at candidate genes, e.g. the *POLL *gene, is a possible explanation for this pattern. The potential impact of selective breeding by man on cattle populations is discussed in the context of selection effects. Our results also suggest that a practical approach to detect loci under selection is to simultaneously apply multiple neutrality tests based on different assumptions and estimations.

## Background

Expectation of neutrality regarding the mutation-drift equilibrium for microsatellite variation is not always valid due to demographic changes, including genetic bottlenecks and admixture (e.g. [1,2]), and selection at linked sites (e.g. [3,4]). In contrast to demographic processes, which affect the entire genome, selection operates at specific sites associated with phenotypic traits, such as important quantitative trait loci (QTLs) and candidate genes. Selection leaves its signature in the chromosomal regions surrounding the sites, where significantly reduced or elevated levels of genetic variation can be maintained at linked neutral loci. Thus, selection not only affects the selected sites but also linked neutral loci and the footprints of selection acting on specific functional loci can be detected by genotyping polymorphic microsatellites in the adjacent non-coding regions [5].

Different statistical methods have been developed to identify outlier loci under the influence of selection [6-13] and adaptations have been attempted to improve the original methods of Lewontin and Krakauer [14], which have been criticized because of their sensitivity to population structure and history (e.g. [15]). Nevertheless, recent studies have shown somewhat inconsistent results obtained by applying the above statistical tests to the same data (e.g. [7,12,16,17]). The Lewontin- Krakauer test [14] is the oldest of these multilocus-comparison methods. Broadly speaking, these methods are derived by using one of the two general approaches detailed below. The first approach is to develop methods with Lewontin and Krakauers' original idea and to use the distribution of estimates of genetic differentiation coefficient *F*_ST _and diversity parameters from individual genetic loci to detect the effects of selection, hereafter termed the *F*_ST_-based approach, such as the FDIST program-based method [9], Bayesian regression [12], and population-specific [7] methods. Schlötterer and colleagues have proposed alternative multilocus simulation-based tests that use summary statistics other than *F*_ST_, such as the ln RV [10], the ln RH [6], and the ln Rθ' [13] tests. These tests involve considering the idea of a 'selective sweep' that arises from natural and artificial selection, and recent genetic exchanges driven by the selective sweep leave a record or "genetic signature" in the genome covering the selected sites and their linked neutral loci. Given that microsatellite loci associated with a recent selective sweep differ from the remainder of the genome, they are expected to fall outside the distribution of neutral estimates of ln RV, ln RH or ln Rθ' values. As reviewed by [18-20], all the methods have potential advantages and drawbacks, which can be due to different underlying assumptions regarding the demographic and mutational models on which they are based, as well as on uncertainty associated with the robustness of the approaches.

The recent increased availability of large genomic data sets and the identification of a few genes or loci as the targets of domestication or subsequent genetic improvement in cattle have renewed the investigation of the genomic effects of selection. Candidate genes and QTL have been described on both BTA1 [21-25] and BTA 20 [26]. On BTA1, the *POLL *gene, characterized by two alleles: *P *(polled) dominant over *H *(horn), is responsible for the polled (i.e. hornless) and horn phenotypes in cattle and has been subjected to both natural and artificial selection. Georges et al. [21] have demonstrated genetic linkage between the *POLL *gene and two microsatellites, *GMPOLL-1 *and *GMPOLL-2*. These loci are syntenic to the highly conserved gene for superoxide dismutase 1 (*SOD1*). In addition, in various breeds the *POLL *gene has been found to be linked to the microsatellites *TGLA49*, *AGLA17*, *INRA212 *and *KAP8*, located in the centromeric region of BTA1 close to the *SOD1 *locus [22,23,25]. To date, on BTA20 several QTL and candidate genes have been reported e.g. growth hormone and prolactin receptor genes [27] affecting conformation and milk production traits, such as body depth (e.g. [28]), udder (e.g. [29]), udder attachment (e.g. [30]), milk yield (e.g. [31]), fat percentage (e.g. [28]), and especially protein content (e.g. [28-30]).

In this study on *Bos taurus*, we present microsatellite data using a relatively larger number of loci than previously reported, which mainly included the 30 microsatellite markers recommended by the International Society for Animal Genetics (ISAG)/Food and Agriculture Organization of the United Nations (FAO) working group (e.g. [2,24]; but see also [32]). Among the 51 microsatellites genotyped on 10 representative cattle populations of different origins (native and modern commercial) and horn statuses (polled and horned) in the northern territory of the Eurasian subcontinent, seven were on BTA1 and 16 on BTA20. We applied four tests to detect molecular signatures of selection, ranging from tests for loci across populations and the recently proposed pairwise population tests using a dynamically adjusted number of linked microsatellites [13]. We compared the consistency of the different neutrality tests available to identify loci under selection in the north Eurasian cattle populations investigated here.

## Materials and methods

### Population samples and genetic markers

Microsatellite data from 10 different cattle (*Bos taurus*) populations including 366 individuals were analyzed. Finnish populations were represented by Finnish Ayrshire (modern commercial, horned, *n *= 40), Finnish Holstein-Friesian (modern commercial, horned, *n *= 40), Eastern Finncattle (native, mostly polled, *n *= 31), Western Finncattle (native, mostly polled, *n *= 37), and Northern Finncattle (native, mostly polled, *n *= 26). We were able to inference the heterozygotic status at the *POLL *locus in 19 phenotypically polled cattle of the three Finnish native populations, on the basis of their offspring/parent phenotypes. In addition, there were 19 animals horned (recessive homozygotic) in the Finnish native populations. Istoben (native, horned, *n *= 40), Yakutian (native, horned, *n *= 51), and Kholmogory (native, horned, *n *= 32) cattle were sampled in Russia. Ukrainian Grey (native, horned, *n *= 30) and Danish Jersey (modern commercial, horned, *n *= 39) were sampled in Ukraine and Denmark, respectively. During sample collection, the pedigree information and the herdsman's knowledge were used to ensure the animals were unrelated. Additional information on these populations has been reported in previous publications [2,33].

Genotypes of the 51 microsatellites were used (for details on the microsatellites, see [33-35]) among which data of the 30 markers from the panel of loci recommended for genetic diversity studies in cattle http://www.projects.roslin.ac.uk/cdiv/markers.html were taken from the literature [2]. The 23 microsatellites (21 new ones and two from the recommended panel) on BTA1 and BTA20 were chosen on the basis of their vicinity to genes and QTL, which could be considered as candidate loci for selection because of their assumed involvement in the polled/horned phenotype [22] and in milk yield and body composition [35]. Details of the primers and microsatellite analysis protocols can be found in CaDBase http://www.projects.roslin.ac.uk/cdiv/markers.html and [34]. In this study, GHRJA.UP, 5'-GGTTCGTTATGGAGGCAATG-3', and GHRJA.DN, 5'-GTCACCGCTGGCAGTAGAT-3' primers were designed based on the sequence of the promoter region of the growth hormone receptor gene [35] containing microsatellite GHRJA. Danish Jersey animals were analyzed only at 41 loci (see Table 1). A full list of the loci studied and their chromosomal and genomic locations, as well as population and basic statistics, are available in Table 1.

**Table 1:** Summary of the microsatellites and basic population genetic estimates for the microsatellites

Locus	BTA	Genomic position (bp)		***A***_**R**_	***H***_**E**_	***F***_**IS**_	FDIST2 test		Ewens-Watterson test			
						
		starts	ends				***F***_**ST**_	*P*	***F***_**OBS**_	***F***_**EXP**_	***P***_**H**_	***P***_**E**_
AGLA17	1	641402	641615	1.37	0.08	-0.049	0.017	0.010**	0.907	0.754	0.978*	0.976*
DIK4591	1	1704734	1705228	2.60	0.32	0.064	0.128	0.660	0.467	0.442	0.844	0.622
DIK1044	1	2829429	2829737	4.86	0.70	0.015	0.118	0.631	0.324	0.329	0.136	0.243
SOD1	1	2914373	2915349	4.78	0.65	0.083	0.173	0.968*	0.331	0.379	0.037*	0.047*
DIK5019	1	3900549	3900808	5.42	0.59	0.190	0.164	0.954*	0.381	0.380	0.005**	0.008**
BMS2321	1	10949260	10949302	3.58	0.45	0.154	0.094	0.410	0.429	0.486	0.424	0.052
BM1824	1	122531990	122532171	3.95	0.72	-0.083	0.122	0.655	0.450	0.487	0.030*	0.231
TGLA304	20	11460907	11460992	3.30	0.49	0.113	0.114	0.573	0.497	0.531	0.237	0.238
BMS1754	20	18439757	18439877	3.47	0.58	0.014	0.094	0.384	0.503	0.536	0.153	0.126
NRDIKM033	20	15598470	15598176	5.20	0.75	-0.004	0.098	0.372	0.234	0.213	0.415	0.466
ILSTS068	20	21675187	21675451	2.07	0.25	0.095	0.146	0.760	0.734	0.751	0.383	0.223
TGLA126	20	21808628	21808745	6.27	0.71	-0.009	0.079	0.170	0.493	0.443	0.085	0.057
BMS2461	20	25278607	25278662	4.83	0.62	0.028	0.180	0.985*	0.227	0.246	0.453	0.760
BMS1128	20	26364064	26364112	3.54	0.52	0.032	0.109	0.534	0.472	0.446	0.503	0.203
BM713	20	26977228	26977280	3.36	0.62	-0.074	0.162	0.907	0.439	0.486	0.197	0.674
DIK2695	20	30452613	30452786	3.60	0.58	-0.027	0.075	0.186	0.432	0.411	0.565	0.274
TGLA153	20	31240022	31240154	4.64	0.71	0.025	0.109	0.521	0.345	0.353	0.101	0.269
GHRpromS	20	31023202	31023306	3.12	0.43	0.006	0.114	0.581	0.426	0.446	0.726	0.268
BMS2361	20	34597279	34597368	5.10	0.72	0.019	0.125	0.698	0.329	0.351	0.045**	0.017**
DIK4835	20	35915540	35916040	4.96	0.65	0.022	0.136	0.788	0.293	0.329	0.252	0.046
AGLA29	20	3842995	38843142	5.49	0.78	-0.006	0.087	0.202	0.363	0.412	0.000**	0.000**
BMS117	20	40015465	40015564	3.88	0.67	-0.018	0.078	0.197	0.377	0.376	0.398	0.272
UMBTL78	20	40177064	40177157	4.22	0.58	-0.033	0.102	0.462	0.298	0.256	0.884	0.229
BM2113	2	88476	88616	5.44	0.79	-0.052	0.119	0.673	0.353	0.379	0.003**	0.005**
INRA023	3	35576043	35576259	4.85	0.70	0.009	0.113	0.564	0.309	0.306	0.238	0.107
ETH10	5	55333999	55334220	4.57	0.67	0.002	0.134	0.789	0.432	0.446	0.049*	0.031*
ETH152	5	NA	NA	4.56	0.71	0.012	0.081	0.171	0.425	0.486	0.008**	0.020
ILSTS006	7	86555402	86555693	5.14	0.77	-0.007	0.076	0.110	0.331	0.351	0.032*	0.057
HEL9	8	NA	NA	5.04	0.70	0.020	0.134	0.792	0.262	0.289	0.240	0.245
ETH225	9	8089454	8089601	5.02	0.71	0.013	0.113	0.560	0.410	0.478	0.009**	0.009**
MM12	9	NA	NA	7.76	0.67	0.017	0.123	0.671	0.312	0.347	0.244	0.112
ILSTS005	10	93304132	93304315	2.17	0.43	-0.026	0.083	0.356	0.686	0.664	0.358	0.390
CSRM60	10	70549981	70550081	7.03	0.72	0.011	0.073	0.094	0.405	0.418	0.046*	0.038*
HEL13	11	NA	NA	3.14	0.51	0.081	0.125	0.678	0.402	0.407	0.529	0.564
INRA032	11	49569411	49569592	3.81	0.62	-0.010	0.142	0.812	0.511	0.537	0.063	0.016
INRA037	11	70730695	70730819	4.54	0.58	0.030	0.129	0.717	0.266	0.243	0.830	0.462
INRA005	12	71751518	71751656	3.18	0.56	0.032	0.088	0.321	0.594	0.596	0.114	0.096
CSSM66	14	6128576	6128773	5.91	0.74	0.002	0.137	0.873	0.312	0.352	0.000**	0.003**
HEL1	15	NA	NA	3.99	0.67	0.020	0.072	0.138	0.468	0.445	0.119	0.155
SPS115	15	NA	NA	5.40	0.58	0.039	0.096	0.416	0.478	0.482	0.228	0.146
INRA035	16	62926476	62926577	2.72	0.23	0.391	0.072	0.266	0.521	0.488	0.746	0.421
TGLA53	16	22214785	22214925	12.25	0.74	0.071	0.099	0.354	0.195	0.213	0.063	0.037
ETH185	17	36598852	36599086	8.31	0.68	0.039	0.146	0.877	0.336	0.303	0.186	0.196
INRA063	18	37562469	37562645	3.31	0.57	0.031	0.110	0.546	0.537	0.487	0.270	0.135
TGLA227	18	60360145	60360234	10.71	0.82	0.005	0.076	0.075	0.282	0.315	0.005**	0.012*
ETH3	19	NA	NA	4.44	0.65	0.009	0.135	0.787	0.407	0.406	0.073	0.139
HEL5	21	11850292	11850455	4.64	0.66	0.038	0.151	0.903	0.424	0.410	0.023*	0.104
TGLA122	21	50825795	50825936	11.36	0.74	0.007	0.069	0.065	0.210	0.213	0.538	0.152
HAUT24	22	45733839	45733962	7.09	0.70	0.025	0.143	0.861	0.406	0.424	0.004**	0.027*
BM1818	23	35634770	35635033	4.03	0.63	0.019	0.102	0.458	0.538	0.486	0.144	0.013*
HAUT27	26	26396836	26396987	8.85	0.61	0.126	0.103	0.453	0.376	0.396	0.083	0.003**

BTA, *Bos taurus *autosome; *A*_R_, allelic richness; *H*_E_, expected heterozygosity, *F*_IS_, inbreeding coefficient, observed homozygosity, *F*_OBS_, and expected homozygosity, *F*_EXP_, NA, not available; the probabilities for the Ewens-Watterson test were calculated based on homozygosity (*P*_H_) or Fishers's exact test (*P*_E_); *, the significance level of *P *< 0.05, **, the significance level of *P *< 0.01; the genomic positions for the loci are BLASTed against STS or primer sequence in ENSEMBL cow genome Btau4.0 http://www.ensembl.org/Bos_taurus/Info/Index updated until 11/02/2010

### Microsatellite variability measures and test for linkage disequilibrium

Microsatellite variability, expected heterozygosity (*H*_EXP_), allelic richness (*A*_R_), and Weir and Cockerham's *F*_ST _[36], were estimated with the FSTAT program, version 2.9.3.2 [37].

The *D*' metric used to estimate the LD was calculated using Multiallelic Interallelic Disequilibrium Analysis Software (MIDAS; [38]). Values of *D*' were calculated for all syntenic marker pairs on BTA1 and BTA20 across the populations. A more detailed description of the estimation of *D*' can be found in [39]. The statistical significance of the observed association between pairs of alleles under the null hypothesis of random allelic assortment was tested using a Monte-Carlo approximation of Fisher's exact test as implemented in the software ARLEQUIN [40] using a Markov chain extension to Fisher's exact test for *R *× *C *contingency tables [41]. A total of 100 000 alternative tables were explored with the Markov chain and probabilities were typically estimated with a standard error of < 0.001. Estimation of the *D*' metric for LD and tests for their significance were conducted only in three Finnish native breeds, i.e. Northern Finncattle, Eastern Finncattle and Western Finncattle. The graphic summary of the significance of LD determinations was displayed using the HaploView program, version 4.0 [42]. Fisher's exact tests in the GENEPOP v 4.0 [43] were applied to assess LD determinations between all locus pairs across the sample.

### Tests to detect loci under selection across populations

Possible departures from the standard neutral model of molecular evolution - potentially revealing demographic events or the existence of selective effects at certain loci - were examined for each locus using the Ewens-Watterson test [44,45] and the Beaumont and Nichols's modified frequentist method [9], as well as a more robust Bayesian test [12].

The Ewens-Watterson test of neutrality was performed with the ARLEQUIN program [40] assuming an infinite allele mutation model. To obtain sufficient precision with this test, the probability was recorded as the mean of 20 independent repeats of 1,000 simulations. The frequentist method used was that proposed by [9], further developed by [12], and implemented in the FDIST2 program http://www.rubic.rdg.ac.uk/~mab/software.html, a currently distributed version of the original FDIST program as described by [12]. FDIST2 calculates *θ*, Weir & Cockerham's [36] estimator of diversity for each locus in the sample. Coalescent simulations are then performed to generate data sets with a distribution of *θ *centered on the empirical estimates. Then, the quantiles of the simulated *F*_ST _within which the observed *F*_ST_'s fell and the *P*-values for each locus were determined. Initially an island model of population differentiation was used and the procedure repeated 50,000 times to generate 95% confidence intervals for neutral differentiation and to estimate *P*-values for departure of the loci from these expectations. Simulation parameters were under an infinite allele mutation model for 100 demes, 10 sample populations, sample sizes of 100, and a weighted *F*_ST _similar to the trimmed mean *F*_ST _calculated from the empirical distribution. Computed by removing the 30% highest and lowest *F*_ST _values observed in the empirical data set, the trimmed mean *F*_ST _is an estimate of the average "neutral" *F*_ST _value uninfluenced by outlier loci (see [46]). This method provides evidence for selection by looking for outliers with higher/lower observed *F*_ST _-values, controlling for *P*-values [12]. The approach is fairly robust regarding variation in mutation rate between loci, sample size, and whether populations are at equilibrium or not [9].

Beaumont & Balding's [12] hierarchical-Bayesian method was performed using the BAYESFST program http://www.reading.ac.uk/Statistics/genetics/software.html package, which generates 2,000 Markov chain Monte Carlo (MCMC) simulated loci on the basis of the distribution of *F*_ST _given the data. The method combines information over loci and populations in order to simultaneously estimate *F*_ST _at the *i*^th ^locus and the *j*^th ^population, *F*_ST_(*i*, *j*), for all *i *loci and *j *populations. A hierarchical model is implemented for *F*_ST_(*i*, *j*) as

$$F_{ST}(i,j)=\frac{\exp(\alpha_{i}+\beta_{i}+\gamma_{ij})}{1+\exp(\alpha_{i}+\beta_{i}+\gamma_{ij})}$$

where α_i_, β_j _and γ_ij _are locus, population and locus-by-population parameters, respectively [12]. In this study, the interpretations of the potential outliers are based on the locus effect (*α*_i_). Outliers from our data set were identified on the basis of the distribution following [12]. Rather than a fixed *F*_ST _as assumed in the above frequentist method of [9], this BAYESFST test uses more information from the raw data and does not assume the same *F*_ST _for each population [5,12].

### Tests to detect loci under selection for pairwise populations

To test for additional evidence of selection, we used the combination of statistics lnRH, lnRV and lnRθ' in the population pairwise comparisons. The principle behind these tests is that variability at a neutral microsatellite locus is given by θ = 4 *N*_e_*μ*, where *N*_e _is the effective population size and *μ *is the mutation rate. A locus linked to a beneficial mutation will have a smaller effective population size and consequently a reduction in variability below neutral expectations. The relative variance in variability, lnRθ, can be assessed instead by estimating the relative variance in repeat number, lnRV, or heterozygosity, lnRH, for loci between populations. The lnRV was calculated using the equation lnRV = ln (*V*_pop1_/*V*_pop2_) where *V*_pop1 _and *V*_pop2 _are the variance in repeat number for population 1 and population 2, respectively [10]. The lnRH test is based on the calculation of the logarithm of the ratio of *H *for each locus for a pair of populations as follows

$$\ln\text{RH}=\ln\frac{{\left(\frac{1}{1-H_{\text{pop1}}}\right)}^{2}-1}{{\left(\frac{1}{1-H_{\text{pop2}}}\right)}^{2}-1}$$

where *H *denotes expected heterozygosity (see equation 2 in [6]). In addition, we attempted to calculate ln Rθ by estimating θ directly using a coalescence-based Bayesian Markov chain Monte Carlo simulation approach employing the MSVAR program [47].

The tests have been shown to be relatively insensitive to mutation rate, deviation from the stepwise mutation model, demographic history of population and sample size [16]. As suggested by [48], to detect the most recent and strong selective sweeps, the combination of lnRH and lnRV statistics is as powerful as lnRV alone, but using both statistics together lowers the rate of false positives by a factor of 3 because the variance in repeat number and the heterozygosity of a population measure different aspects of the variation at a locus. Thus, combinations of any two of the three tests were implemented here and significance of lnRH, lnRV and lnRθ' for each comparison was calculated according to standard methods [6,10,48]. These statistics are generally normally distributed, and simulations have confirmed that outliers (e.g. more than 1.96/2.58 standard deviations from the mean for 95%/99% confidence intervals, respectively) are likely to be caused by selection [48]. The tests were implemented for every pairwise comparison involving native populations from different trait categories (Eastern Finncattle, Western Finncattle and Northern Finncattle vs. Yakutian, Istoben, Kholmogory and Ukrianian Grey), i.e. 12 population pairs for the horn (polled/horned) trait.

### Tests to detect loci under selection within a population

The coalescence simulation approach using the DetSel 1.0 program [49] was used to detect outlier loci within the Finnish native populations (Eastern Finncattle, Western Finncattle and Northern Finncattle). It has the advantage of being able to take into account a wide range of potential parameters simultaneously and giving results that are robust regarding the starting assumptions. For each pair of populations (*i*, *j*), and for all loci, we calculated *F*_i _and *F*_j _(*F*_i _and *F*_j _are the population-specific divergence; for details see [7,49]) and generated the expected joint distribution of *F*_i _and *F*_j _by performing 10,000 coalescent simulations. Thus, every locus falling outside the resulting confidence envelope can be seen as potentially under selection. The following nuisance parameters were used to generate null distributions with similar numbers of allelic stages as in the observed data set: mutation rates (infinite allele model) *μ *= 1 × 10^-2^, 1 × 10^-3^, and 1 × 10^-4^; ancestor population size *N*_e _= 500, 5,000, and 50,000; times since an assumed bottleneck event *T*_0 _= 50, 500, and 5,000 generations; time since divergence *t *= 50 and 500; and population size before the split *N*_0 _= 50 and 500. In order to detect outlier loci potentially selected for the polled trait within the three Finnish native cattle populations, the DetSel program was run for comparison between the two subpopulations representing the definitely polled (*n *= 19) and horned (*n *= 19) animals, respectively.

## Results

### Genetic diversity and differentiation

A complete list of loci and their variability in the 10 cattle populations are shown in Table 1. The overall genetic differentiation across loci was 0.117 (*F*_ST _= 0.117, 95% CI 0.108 - 0.125). *F*_ST _values for an individual locus varied from 0.017 (SD = 0.011) at *AGLA17 *on BTA1 to 0.180 (SD = 0.057) at *BMS2461 *on BTA20. Mean population differentiations for loci on BTA1 and BTA20 were 0.126 (*F*_ST _= 0.126, 95% CI 0.103 - 0.143) and 0.118 (*F*_ST _= 0.118, 95% CI 0.100 - 0.139), respectively. Neither of the values indicated significant difference from the average for loci on other chromosomes (*F*_ST _= 0.114, 95% CI 0.104 - 0.124).

Levels of variation across populations, including allelic richness (*A*_R_) and expected heterozygosity (*H*_E_), were in similar ranges as for microsatellites on BTA1, BTA20 and other autosomes, with the smallest variations observed at *AGLA17 *(*A*_R _= 1.37, *H*_E _= 0.08). The highest *H*_E _of 0.79 was observed at *BM2113 *(BTA2) and the highest *A*_R _of 11.36 at *TGLA122 *(BTA21). Most *F*_IS _values were positive and for some loci significantly positive. Of the 13 negative *F*_IS _values, seven occurred for loci on BTA20, and two for loci on BTA1. Loci on BTA1 and BTA20 did not show a significant reduction or increase in mean *F*_IS _compared with the loci on other autosomes (other bovine autosomes, mean *F*_IS _= 0.038; BTA1, mean *F*_IS _= 0.053, Mann-Whitney test *U *= 118, *P *= 0.409; BTA20, mean *F*_IS _= 0.011, Mann-Whitney test *U *= 273.5, *P *= 0.227). Given the range of observations of *F*_IS _at an individual locus, there were no marked difference among the three classes of loci (BTA1, -0.083 - 0.190; BTA20, -0.074 - 0.113; other BTAs, -0.052 - 0.391).

### Linkage disequilibrium

**Figure 1:**
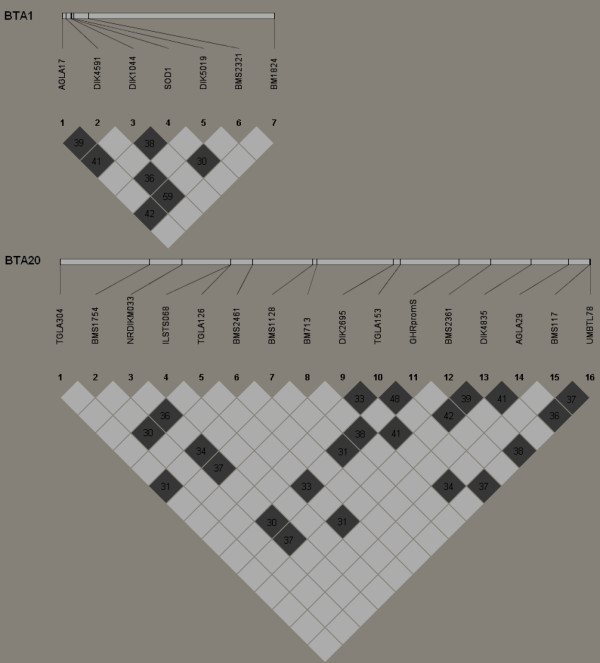
**Detailed view of the extent and significance of LD in the cattle populations using the Haploview 4.0 program**. Numbers in the blocks indicate the percentage of the LD metric *D*' values > 0.3; shadings indicate Fisher's exact test significance levels: white, *P *> 0.05; light shading, *P *< 0.05.

The strength of pairwise linkage disequilibrium (LD) between markers was estimated and the average *D*' value of pairwise syntenic markers was 0.32 across BTA1 and 0.28 across BTA20, both of which are significantly (*P *< 0.05) higher than for non-syntenic markers (0.15; only the *D*' > 0.3 are shown in Figure 1). Figure 1 also shows matrices of LD significance levels for all possible locus combinations of the loci on BTA1 or BTA20 in their chromosomal order. Of the 120 pairwise comparisons of the 16 loci on BTA20, a total of 22 (22/120, 18.3%) tests showed *P *values below 0.05. Likewise, LD between markers on BTA1 provided seven (7/21, 33.3%) significant observations. However, a substantially smaller proportion (34/1124, 3.0%) of significant (*P *< 0.05) pairs was found between non-syntenic markers. In general, significantly higher levels of LD were observed for syntenic markers on BTA1 and BTA20 than that for non-syntenic markers. There was no evidence of LD blocks on either of the chromosomes.

### Evidence for selection across the populations

The Ewens-Watterson test enables detection of deviations from a neutral-equilibrium model as either a deficit or an excess of genetic diversity relative to the number of alleles at a locus (see [50]). When applying the tests for all the microsatellites, we detected 13 loci (*AGLA17*, *DIK5019*, *SOD1*, *AGLA29*, *BMS2361*, *BM2113*, *ETH10*, *ETH225*, *CSSM66*, *ETH152*, *TGLA227*, *HAUT24*, and *CSRM60*) on 10 different chromosomes exhibiting significant probabilities for the Ewens-Watterson test based on both homozygosity (*P*_H_) and Fisher's exact test (*P*_E_) (see Table 1). Of the 13 loci, one (*AGLA17*) exhibited a significant (*P *< 0.05) deficit of heterozygosity and all the other 12 loci exhibited a significant (*P *< 0.05) excess in genetic diversity relative to the expected values; these patterns are consistent with directional and balancing selection, respectively. The 12 loci generated average *P *values significantly (Student's *t *test: $\overset{ª}{P_{H}}$ = 0.020, *t *= -5.65, *P *< 0.0001; $\overset{ª}{P_{E}}$ = 0.014, *t *= -5.69, *P *< 0.0001) below than the expected median value of 0.5. However, average *P *values of 0.313 for *P*_H _(*t *= -4.63, *P *> 0.1) and 0.232 for *P*_E _(*t *= -8.69, *P *> 0.1) were observed in the remaining 38 loci which were not under selection. The observation provided further evidence that selection affected genetic diversity at the microsatellites under selection.

**Figure 2:**
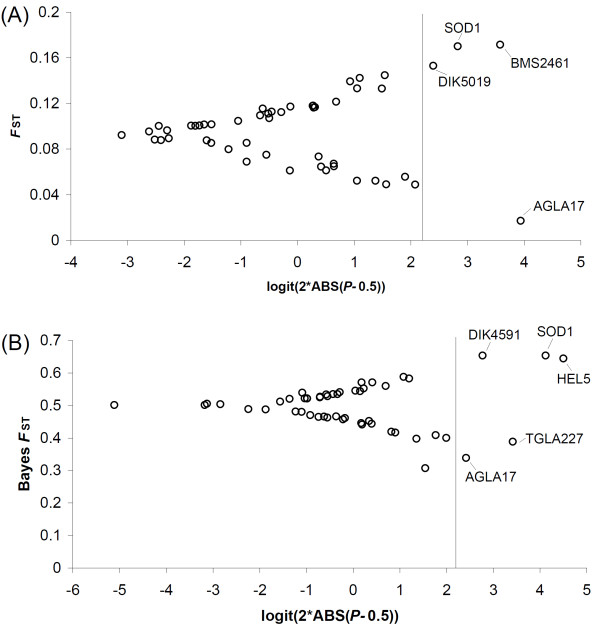
**Results of (A) the FDIST2 and (B) BAYESFST tests**. The solid lines indicate the critical cutoff for the *P*-value at the 0.05 level.

The results of the analyses with the FDIST2 program are presented in Table 1 and Figure 2a. This summary-statistic method, based on simulated and observed *F*_ST _values, identified four loci (*SOD1*, *BMS2461*, *DIK5019 *and *AGLA17*) as outliers showing footprints of selection in the analyses, including all 10 populations, at the 5% significance level. Of the four significant loci, three (*SOD1*, *BMS2461 *and *DIK4519*) with higher *F*_ST _values indicated a sign of directional selection and one locus (*AGLA17*) appearing in the lower tail of the *F*_ST _distribution suggested a signature potentially affected by balancing selection (Figure 2a). In the Bayesian *F*_ST_-test (Figure 2b), which was based on a hierarchical regression model, three loci (*HEL5*, *DIK4591*and *SOD1*) were detected as being directionally selected and two (*AGLA17 *and *TGLA227*) as under balancing selection. Overall, across all the populations, two loci, *AGLA17 *and *SOD1*, exhibited the strongest evidence of selection with all three statistical approaches, which provided good support to their status as outliers due to selection. Two loci (*DIK5019 *and *TGLA227*) exhibited significant departure from the neutral expectations in two out of the three selection tests. Furthermore, 12 loci (*AGLA29*, *BMS2361*, *BM2113*, *ETH10*, *ETH225*, *CSSM66*, *ETH152*, *HAUT24*, *CSRM60, BMS2461, HEL5 *and *DIK4591*) can be regarded as candidates affected by selection, but were revealed only in one of the three tests. Interestingly, according to ENSEMBL cow genome http://www.ensembl.org/Bos_taurus/Info/Index the significant locus *AGLA17 *under balancing selection was about 1.78 cM upstream from the candidate locus for *POLL*, whereas locus *SOD1 *under directing selection was located about 3.87 cM downstream from the candidate locus. It should be noted that the *F*_ST_-based tests of selection are prone to false positives because of sensitivity to demographic history [51], heterozygosity among loci in mutation rate [52] and locus-specific phenomena not related to selection [48]. Nevertheless, we expect the set of loci identified by *F*_ST_-based tests to be enriched for the true positives in further tests.

### Tests for selection for pairwise populations

Since each of the five tests used above relies on somewhat different assumptions, loci that are repeatedly found to be outside the range expected for neutrality are extremely good candidates for markers under selection. Moreover, LD is known to be extremely high for the six BTA1 microsatellites near the candidate gene affecting the presence or absence of horns in *Bos taurus*, thus the region under selection is likely to be quite wide. Despite the possible presence of a few false positives, the full set of seven loci (*SOD1*, *BMS2461*, *DIK5019*, *HEL5*, *DIK4591*, *TGLA227 *and *AGLA17*) was used for further analyses. The lnRθ methods (lnRH, lnRV and lnRθ') use heterozygosity or variance difference, rather than population divergence, to test for selection. Significant results for the lnRθ tests for selective sweeps involve the two loci (*AGLA17 *and *SOD1*) detected by the Ewens-Watterson test and the *F*_ST_-based tests for pairwise combinations (*n *= 12) of three native Finnish cattle populations and four old native populations from Russia and Ukraine (Table 2).

**Table 2:** Estimates of lnRV, lnRH and lnRθ' for the pairwise comparisons

Pairwise comparison	lnRV		lnRH		lnRθ'	
	
	*AGLA17*	*SOD1*	*AGLA17*	*SOD1*	*AGLA17*	*SOD1*
Eastern Finncattle - Istoben	*	*	n.s.	n.s.	*	n.s.
Eastern Finncattle - Yakutian	*	**	*	**	**	*
Eastern Finncattle - Ukrainian Grey	**	**	*	*	**	*
Eastern Finncattle - Kholmogory	*	**	*	*	*	*
Western Finncattle - Istoben	**	*	**	**	*	*
Western Finncattle - Yakutian	**	**	*	*	*	**
Western Finncattle - Ukrainian Grey	*	*	**	*	*	*
Western Finncattle - Kholmogory	*	*	*	*	*	**
Northern Finncattle - Istoben	*	n.s	*	n.s.	n.s.	*
Northern Finncattle - Yakutian	*	n.s.	n.s.	*	n.s.	n.s.
Northern Finncattle - Ukrainian Grey	**	*	n.s.	n.s.	n.s.	n.s.
Northern Finncattle - Kholmogory	*	n.s.	n.s.	*	n.s.	n.s.

* Significance *P *< 0.05, ** *P *< 0.01, n.s., not significant

Significant results for selective sweeps at loci *AGLA17 *and *SOD1 *were obtained for 12 pairwise population comparisons for each of the three different measures of lnRθ (Table 2). Of the pairwise comparisons, a total of 28 and 26 significant (*P *< 0.05) or very significant (*P *< 0.01) results were observed at *AGLA17 *and *SOD1*, respectively, in the three tests. Both loci (*AGLA17 *and *SOD1*) appeared in all three different measures of lnRθ for eight or more comparisons (Table 2), that is, lnRθ (lnRH, lnRV and lnRθ') values deviating by more than 1.96 standard deviations from the mean. Accordingly, the pairwise comparisons between either of Eastern Finncattle and Western Finncattle and populations of Yakutian, Kholmogory and Ukrainian Grey were significant for all three estimators. All the comparisons between populations yielded at least two significant results for the three estimators. In total, 54 (75% 54/72) significant comparisons involved *AGLA17 *or *SOD1 *in the comparisons between Finnish native populations (Northern Finncattle, Eastern Finncattle and Western Finncattle) vs. the native populations from Russia and Ukraine (Istoben, Ukrainian Grey, Kholmogory and Yakutian Cattle), which suggested that selective sweeps had taken place in the Finnish native populations.

### Tests for selection within the Finnish native populations

**Figure 3:**
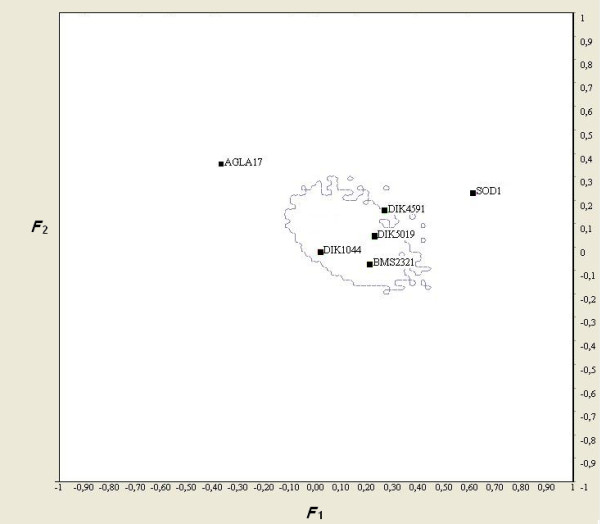
**Pairwise comparison of Finnish native cattle populations performed with DetSel**. The test was at the 95% confidence envelope: plot of *F*_2 _against *F*_1 _estimates for the subpopulation pair polled vs. horned.

The coalescent simulation, which was based on a population split model [49], was performed with the DetSel program within the Finnish native populations with very similar demographical backgrounds (Eastern Finncattle, Northern Finncattle and Western Finncattle). Among the six BTA1 microsatellites around the candidate loci, all are polymorphic in the three populations involved in the pairwise-subpopulation comparison. In the pairwise comparison between definitely polled (*n *= 19) and horned (*n *= 19) cattle, loci *AGLA17 *and *SOD1 *were significantly outside the 99% confidence interval (Figure 3), while locus *DIK4591 *fell slightly outside the 95% confidence envelope in the three comparisons, which are thus considered as false positives, i.e., the locus was detected as an outlier because of the 5% type I error. The outlier behavior for loci *AGLA17 *and *SOD1 *was deemed to be the result of strong local effects of hitchhiking selection.

## Discussion

In this study, besides 28 microsatellites on other cattle autosomes used as a reference set of markers, seven microsatellites on BTA1 and 16 on BTA20 around candidate loci were screened for the footprints of selection among 10 cattle populations with divergent horn or production traits. Across different statistical analyses, a highly divergent pattern of genetic differentiation and large differences in levels of variability were revealed at the loci *SOD1 *and *AGLA17 *among populations, which was inconsistent with neutral expectations. The results indicated divergent 'selective sweeps' at *AGLA17 *and *SOD1*, probably caused by selection of the closely-linked candidate loci for the horned/polled trait, e.g. the *POLL *gene.

### Evidence of selection of microsatellites surrounding the *POLL *gene

Because revealing outlier loci in genome scans currently depends on statistical tests, one of the main concerns is to highlight truly significant loci while minimizing the detection of false positives [44]. Using a multilocus scan of differentiation based on microsatellite data, we compared three different methods that aimed at detecting outliers from simulated neutral expectations: 1) the Ewens-Watterson method [44,45], 2) the FDIST2 method [9], and 3) a BAYESFST method [12]. Outliers were identified for 15 loci using a 5% threshold, which was robust across methods for two loci (*SOD1 *and *AGLA17*). The locus *SOD1 *presented a higher differentiation (*F*_ST _value) than expected, suggesting that it could have been affected by the action of diversifying selection among homogeneous gene pools and populations. In contrast, the locus *AGLA17 *presented a lower genetic differentiation than expected, which could represent signatures of homogenizing selection among populations and/or balancing selection within populations. All three methods identified loci *SOD1 *and *AGLA17 *as good candidates for selection on the polled trait. However, several significant loci were detected only by one or two of the tests and thus could not be accepted as reliable outliers with the remaining tests. The results obtained by the three methods are not totally consistent, probably because of the difference in statistical power using multiple measures of variability, each of which measures different parameters and relies on different assumptions, e.g. heterozygosity and variance in allele size [48], as detailed in e.g. [53-55].

Besides the global analyses, detection of outlier loci was also done using pairwise analyses. This helped to reveal loci with a major overall effect as well as loci responding with different strengths to artificial selection on the individual populations. Among the population chosen for the pairwise analyses, the lnRθ (lnRV, lnRH and lnRθ') tests yielded a high number of significant (*P *< 0.05) results at *SOD1 *and *AGLG17 *according to the three estimators of lnRθ (Table 2). This finding conforms well to the previous results of selective sweeps associated with hitchhiking selection with one or more genes with locally beneficial mutations. Although there is difference in the statistical power to detect selection, as discussed in [6,48,56], the three estimators of lnRθ provide additional robust evaluation of potential selective sweeps for the pairwise population comparisons.

Neutrality tests for microsatellites focus mainly on unlinked loci and are based on either population differentiation (*F*_ST_) or reduced variability (lnRθ). Our proposed tests consider lnRθ of several linked loci for the inference of selection. While the single-locus lnRθ-test is largely independent of the demographical past, the additional power of linked loci is balanced by the cost of an increasing dependence of the demographic past due to the fact that LD is extremely sensitive to the demographic history. Thus, pairwise analyses between sub-populations may decrease the demographic effects in accounting for the selection. As indicated in Figure 3, the great majority of loci always fall in the confidence region of the conditional pairwise-subpopulation distributions of branch length estimates, while some loci do not. Overall, we identified two loci (*SOD1 *and *AGLA17*) that were probably subject to selection in the three Finnish native populations. Thus, we concluded that the distribution of variability at these loci could have been shaped by forces other than demographic effects e.g. genetic drift. Although the locus *DIK4591 *was located on the edge or fell just outside the high probability region of the expected conditional distribution in the Finnish native populations, we must be cautious about the locus because the estimation of *F*_i _parameters is discontinuous as a result of the discrete nature of the data, i.e. the allele counts (e.g. [7]). However, it is worth noting that not all significant loci detected by other methods could be accepted as trustworthy outliers with DetSel due to technical constraints, which means that if a locus is monomorphic in one population of the pair analyses with DetSel are not possible.

Tests to detect outlier loci that deviate from neutral expectation cannot identify false positives (type I errors). Thus, we conducted the three different neutrality tests (the Ewens-Watterson test, the FDIST test and the BAYESFST test), setting a 95% *P *level criterion to identify loci under selection pressure, at which the expected number of false positive loci is 51 × 0.05 = 2.55. We still found 13, four and five outlier loci, respectively, indicating that at least some of the outlier loci are unlikely to be false positives. As suggested by [5], a practical approach to strengthen the candidate status of identified outlier loci is to apply two or more neutrality tests simultaneously based on different assumptions and parameter estimation and only consider outlier loci that are supported by several methods for subsequent validation steps. Thus, the fact that some loci are identified by one neutrality test, but not by others, suggests that their status as candidate loci under selection must be regarded with considerable caution. However, significant deviations from neutrality expectation using multiple tests do not necessarily mean that a particular locus has been affected by hitchhiking selection. In this case, we applied three different pairwise population neutrality tests in 12 separate comparisons using two loci (across the populations: 3 × 12 × 2 = 72 separate tests). This is expected to result in approximately four false positives at the 95% *P *level. The fact that we observed as many as 54 deviations (Table 2) at the 95% *P *level indicates that it is unlikely that all the outliers identified by pairwise analyses are due to type I errors. Moreover, no locus showed only one significant deviation in one pairwise population comparison (see Table 2). Therefore, it can be considered that the approach was quite robust and conservative in the detection of the effects of hitchhiking selection, particularly when additional pairwise analyses were applied.

### Interpretation of the outlier loci and caveats

Actually microsatellites are unlikely to be the target of selection, but are merely tightly linked to the candidate genes. Since the microsatellites used are located close to some functional candidate genes (or QTLs) on the same chromosome, this indicates a high probability that one or several good candidate genes (or QTLs) is/are tightly linked to some of the microsatellites. In many of the cases examined to date, selective sweeps have affected only a very small region, potentially containing only one or a few genes, except in the case of extremely strong selection (see [57]). Empirical studies indicate that the negligible LD between a hitchhiking locus and a candidate gene underlying selection varied from tens of bp (e.g. [55]) to tens or even hundreds of kb (see [58,59]), which depends on a variety of factors such as the genomic regions (e.g. sex chromosome vs. autosome) and populations (e.g. domesticated vs. wild) investigated, and the type of markers (e.g. EST- or MHC-microsatellites vs. microsatellites) used. It has also been suggested that the LD between loci and candidate genes affected by selection is determined mainly by the strength of selection, local recombination rate, population history, and the age of the beneficial allele [60]. Whatever the reason, significant LD was detected with inter-marker genomic distances between *ca*.1100 kb and *ca*.10300 kb in this study (see Figure 1), a considerably wider interval than reported previously.

We detected two microsatellite loci (*AGLA17 *and *SOD1*) probably linked to the candidate gene for the polled trait in the populations investigated. The polled trait is an autosomal dominant trait in cattle and to date the genes controlling this trait have not been specifically identified. However, the gene causing the absence of horns is known to be at the centromeric end of BTA1. Several factors have potentially driven evolution of the functionally important candidate locus including artificial selection and mating system. In Finnish native cattle populations, polled animals were particularly favored during selective breeding. However, we did not detect any locus under selection on BTA20 despite that the fact that several microsatellites including GHRJA surround the growth hormone receptor gene. Growth hormone receptor belongs to the large superfamily of class 1 cytokine receptors. It has various roles in growth, lactation and reproduction in cattle and has been identified as a candidate gene affecting a few key quantitative traits. Therefore, it is not specific to dairy traits but to traits related with growth, lactation and reproduction. Among the cattle populations investigated here, no contrasting differences in growth, lactation or reproduction was observed. In addition, a recent study on the evolution of the cytoplasmic domains of the growth hormone receptor gene in Artiodactyla (see [61]) has suggested that possible effects of selective sweeps on growth hormone receptor gene in bovine occurred before domestication and not among the domestic breeds.

Unfortunately, due to the lack of information on the mutation and recombination rates, as well as the effective population size for these data, estimation of the selection coefficient is not possible here (see [59]). Given that the genomic interval of significant LD is comparable with the findings of hitchhiking around two anti-malarial resistance genes in humans [58] and microsatellite hitchhiking mapping in the three-spined stickleback [59], the hitchhiking selection in this genomic region might be fairly strong. Moreover, the availability of genomic resources (e.g. NCBI Bovine Genome Resources; http://www.ncbi.nlm.nih.gov/projects/genome/guide/cow/)in bovine makes it possible to develop more precise approaches with other much more frequent markers such as SNP. Genotyping an additional set of high density SNP between *AGLA17 *and *SOD1 *markers in the populations investigated will definitely give more precise information on selection and LD in the region.

Because the populations studied here are not experimental, they differ for many characteristics other than the polled and horned traits. Thus, some of the genetic differentiation could have been due to other selective forces, e.g. pathogens. In addition, since our data violate at least partly the model assumptions of equal population size and migration rates between populations for the FDIST2 test, the outliers from the test alone should be considered with caution although the multiple neutrality tests based on different assumptions and parameter estimation can minimize the possibility of false positives. Moreover, selection is not the only possibility for changes in the distribution of variation to occur at particular loci, reduced variation or increased differentiation can result from chance alone, e.g. genetic drift, bottlenecks or founder events [57]. To obtain clear evidence for selection of these markers, we must analyze nucleotide variations between polled and horned populations.

## Conclusions

Our microsatellite data from northern Eurasian cattle populations empirically indicate a practical approach for identifying the best candidate loci under hitchhiking selection by simultaneously applying multiple neutrality tests based on different assumptions and parameter estimations. By analyzing microsatellite markers adjacent to functional genes, we identified two loci (*SOD1 *and *AGLA17*) that are "selection candidate" targets associated with the horned/polled trait in cattle. This result could be further confirmed by using a more densely spaced set of markers. It would also be of great interest to see if similar patterns of selection around the *POLL *gene are observed in commercial beef breeds such as Australian Brangus, Angus and Hereford breeds, where dehorning and breeding practices for polled cattle have been an accepted part of cattle management for generations. Another future challenge is to verify the signal of artificial selection on the *POLL *gene, possibly using the next generation sequencing technology to detect the nucleotide variation of the gene between polled and horned cattle. In addition, the approach we have taken in this paper can be easily extended to other cases and marker types. For example, diversity among cattle has been directed by man towards different goals (e.g. draft, milk, meat, fatness, size, color, horn characteristics, behavior, and other characteristics) during many generations of selection. Each of these selection events has potentially left a signature of selection on the genes and their neighboring loci that could be detected by using tests such as we have applied here. As a marker technology, SNP would offer the advantage of higher throughput when scanning the genome for evidence of hitchhiking selection.

## Competing interests

The authors declare that they have no competing interests.

## Authors' contributions

MHL designed the study, performed the data analysis and wrote the manuscript. TI-T did the laboratory work and contributed to the manuscript writing and data analysis. HL did the laboratory work, contributed to the manuscript writing and data analysis. JK planned and coordinated the whole study, and contributed to the manuscript writing. All the authors read and approved the final manuscript.
